# Effects of NiO nanoparticles on the magnetic properties and diffuse phase transition of BZT/NiO composites

**DOI:** 10.1186/1556-276X-7-59

**Published:** 2012-01-05

**Authors:** Parkpoom Jarupoom, Sukum Eitssayeam, Kamonpan Pengpat, Tawee Tunkasiri, David P Cann, Gobwute Rujijanagul

**Affiliations:** 1 Department of Physics and Materials Science, Faculty of Science, Chiang Mai University, Chiang Mai, 50200, Thailand; 2 Materials Science Research Center, Faculty of Science, Chiang Mai University, Chiang Mai, 50200, Thailand; 3 Faculty of Materials Science, Department of Mechanical Engineering, Oregon State University, Corvallis, Oregon, 97331, USA

**Keywords:** ceramics, composites, magnetic properties, electrical properties, microstructure

## Abstract

A new composite system, Ba(Zr_0.07_Ti_0.93_)O_3 _(BZT93) ceramic/NiO nanoparticles, was fabricated to investigate the effect of NiO nanoparticles on the properties of these composites. *M-H *hysteresis loops showed an improvement in the magnetic behavior for higher NiO content samples plus modified ferroelectric properties. However, the 1 vol.% samples showed the optimum ferroelectric and ferromagnetic properties. Examination of the dielectric spectra showed that the NiO additive promoted a diffuse phase transition, and the two phase transition temperatures, as observed for BZT93, merged into a single phase transition temperature for the composite samples.

## Background

Ferroelectric materials are widely used in a broad range of applications, especially in the design of electronic devices such as non-volatile memory, capacitors, transducers, actuators, etc. [1,2]. Barium zirconate titanate (Ba(Zr_x_Ti_1-x_)O_3_) [BZT] is one such interesting ferroelectric material due to its high relative permittivity, which makes it a very attractive material for use in capacitor applications such as boundary layer capacitors and multilayer ceramic capacitors [3-6]. Furthermore, BZT for some compositions exhibits high ferroelectric and piezoelectric properties. Due to the environmental concern, this material is also beneficial since it is a lead-free material.

Recently, much attention has been paid to multiferroic materials because of the coexistence of ferromagnetic and ferroelectric ordering at room temperature. However, multiferroic materials which exhibit both high ferromagnetic and ferroelectric properties are very rare. This is because ferromagnetic materials need transition metals with unpaired 3*d *electrons and unfilled 3*d *orbitals, while ferroelectric polarization requires transition metals with filled 3*d *orbitals [7]. An alternative way to obtain high ferromagnetic and magnetic properties is to produce composite materials which contain combined ferroelectric and magnetic phases. These materials are called multiferroic composites, and many authors have fabricated and reported the properties of multiferroic composites [8]. In this work, a new system of multiferroic composites was fabricated. The BZT in the composition of Ba(Zr_0.07_Ti_0.93_)O_3 _(BZT93) was synthesized and used as matrix for the composites. NiO nanopowder with a particle size of approximately 100 nm was added to BZT93, and the mixed materials were sintered at various sintering temperatures to form the composites. Properties of the composites were then determined and reported.

## Methods

The composites were prepared by a conventional mixed-oxide method. BZT powder was prepared based on the stoichiometric formula Ba(Zr_0.07_Ti_0.93_)O_3_. The raw metal oxide, BaCO_3_, TiO_2_, and ZrO_2 _were mixed and calcined at 1,200°C for 2 h. Different volume ratios (0, 1, 2, and 3 vol.%) of the NiO nanoparticles (Sigma-Aldrich Corporation, St. Louis, MO, USA; with a particle size of < 100 nm) were mixed with the BZT93 powder and then milled for 24 h. The ball-milled powders were pressed into a disk shape and then sintered at temperatures ranging from 1,250°C to 1,450°C for 2 h. The densities of all the disks were determined after sintering using the Archimedes method. Phase formation of the sintered ceramics was investigated by X-ray diffraction [XRD] technique. The magnetic properties were measured using a vibrating sample magnetometer of the Lake Shore Model 7404 (Lake Shore Cryotronics, Inc., Westerville, OH, USA). The ferroelectric properties were performed using a Sawyer-Tower circuit. Relative permittivity and tangent loss were measured as a function of temperature using an LCR meter.

## Results and discussion

### Densification and phase formation

**Figure 1:**
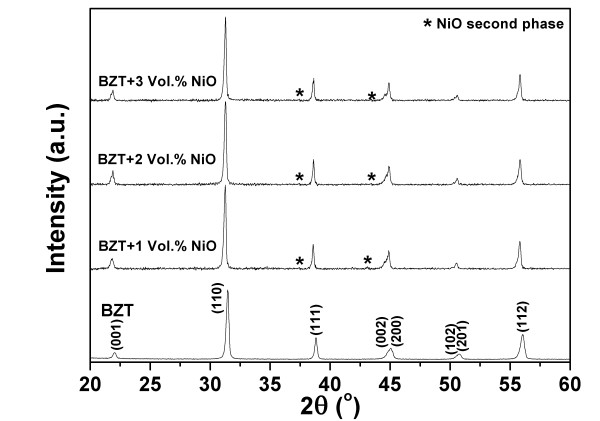
**X-ray diffraction patterns of pure BZT93 and BZT93/NiO composites**.

In this study, a range of sintering temperatures was used to fabricate the tested composites to determine the optimum sintering temperature which provided the optimum properties. For pure BZT93 ceramics, the optimum sintering temperature was 1,450°C, while for the BZT93-NiO composites, 1,300°C. This lower sintering temperature is due to the mismatch between the different components, leading to an inhibition of the sintering ability. The optimum sintering temperature samples were selected for characterization. The phase formation of the pure BZT93 ceramic and composites sintered at an optimum sintering temperature was determined using the XRD technique at room temperature. The XRD results are shown in Figure 1. For the pure BZT93, the XRD pattern corresponded to a pure orthorhombic perovskite phase [9,10]. In the case of the composites, the XRD peaks at 2*θ *~ 37° and 44° indicated an impurity phase. The impurity peaks were identified as NiO, corresponding to the JCPDS file no. 044-1159, confirming a formation of the composites.

### Magnetic and ferroelectric properties

**Figure 2:**
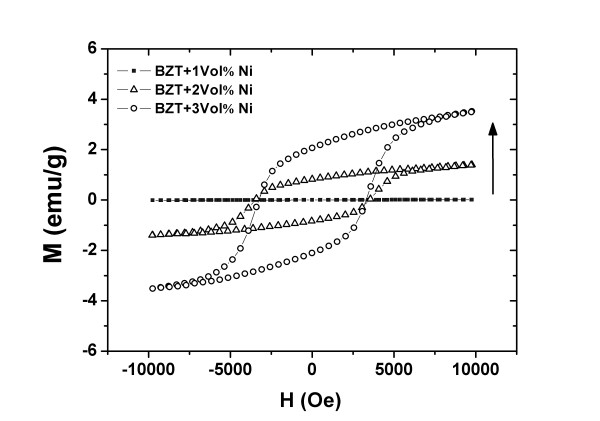
**Magnetization (*M*) vs. applied magnetic field (*H*) of the pure BZT93 ceramic and composites**.

**Figure 3:**
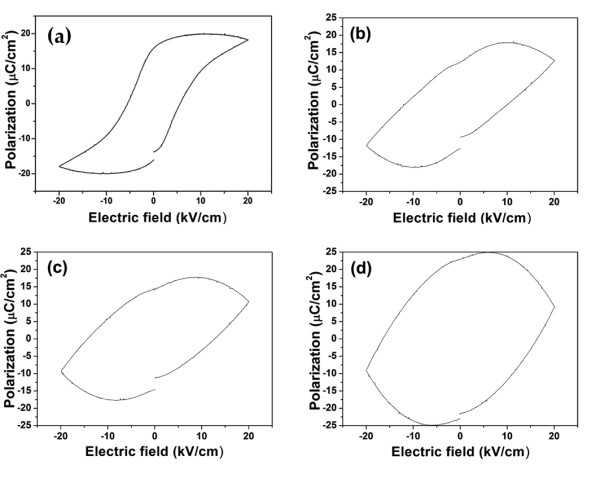
***P-E *hysteresis loops**. (**a**) Pure BZT93, (**b**) BZT93 + 1 vol.% NiO, (**c**) BZT93 + 2 vol.% NiO, and (**d**) BZT93 + 3 vol.% NiO.

Figure 2 shows the *M*-*H *magnetic hysteresis loops of the samples measured at room temperature. The 1 vol.% sample exhibited a weak magnetic behavior. However, an improvement in magnetic properties was clearly observed for the composites containing NiO > 1.0 vol.%. The values of the coercive magnetic field [*H*_c_] and remnant magnetization [*M*_r_] of the samples are listed in Table 1. Figure 3 shows the P-E ferroelectric hysteresis loops (at room temperature) with different NiO contents. The shape of the hysteresis loop for the pure BZT93 ceramics indicates a normal ferroelectric behavior. For samples with higher NiO concentrations, however, the hysteresis loop became more slanted. Furthermore, a lossy capacitor hysteresis loop was clearly observed for the 3 vol.% sample. This may be due to the NiO additive producing a higher electrical conductivity or higher leakage characteristic in the samples. The ferroelectric properties such as remanent polarization [*P*_r_] and coercive field [*E*_c_] are shown in Table 1. Based on the results, the 1 vol.% samples showed the optimum properties combining between the ferroelectric and ferromagnetic properties (*M*_r _= 0.02 emu/g, *H*_c _= 4.51 kOe, *P*_r _= 13.1 μC/cm^2^, and *E*_c _= 9.9 kV/cm) of this composite system. These ferromagnetic and ferroelectric properties were considerably high for single-phase multiferroic materials [11,12] and other multiferroic composites [13,14].

**Table 1:** Unit cell volume, magnetic, and ferroelectric properties of BZT93/NiO composites

NiO (vol.%)	Unit cell volume (Å^3^)	*M*_r_ (emu/g)	*H*_c_ (kOe)	*P*_r_ (μC/cm^2^)	*E*_c_ (kV/cm)	*δ_γ_* (°C)
0	65.05	0	0	15.9	5.7	39.2
1	65.26	0.02	4.51	13.1	9.9	52.5
2	65.30	0.84	3.51	14.8	12.7	53.3
3	65.31	2.8	3.33	23.1	16.4	58.3

*M*_r_, remnant magnetization; *H*_c_, coercive magnetic field; *P*_r_, remanent polarization; *E*_c_, coercive field; *δ_γ_*, diffuseness parameter.

### Dielectric properties and phase transition

**Figure 4:**
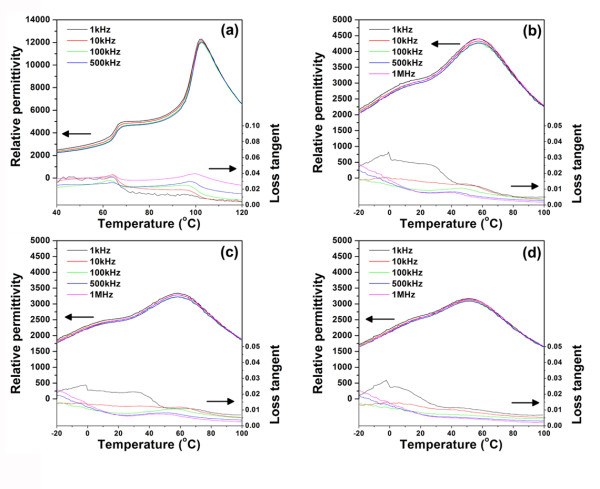
**Relative permittivity and loss tangent as a function of temperature**. (**a**) Pure BZT93 ceramic, (**b**) BZT93 + 1.0 vol.% NiO, (**c**) BZT93 + 2.0 vol.% NiO, and (**d**) BZT93 + 3.0 vol.% NiO.

Figure 4 shows plots of the relative permittivity and loss tangent as a function of temperature at various NiO concentrations. Two phase transition peaks in the permittivity curve were observed for the pure BZT93. The relative permittivity and loss tangent curves for the pure BZT93 ceramic are similar to those reported in a previous work [8,15]. Furthermore, all samples showed a weak frequency dispersion of the relative permittivity. However, an obvious change in the relative permittivity curve was observed when NiO was added to the samples. The transition temperature [*T*_m_] at maximum relative permittivity [*ε*_r, max_] decreased from 105°C for the pure BZT93 ceramics to 60°C for the 1.0 vol.% sample, then gradually decreased to 57°C for the 3.0 vol.% sample. Moreover, the maximum relative permittivity decreased from 12,000 for the pure BZT93 ceramics to 3,200 for the 3.0 vol.% samples. In addition, the two phase transition temperatures merged into a single diffuse phase transition at higher NiO contents (Figure 4d). To check the effect of NiO on the degree of the diffuse phase transition, diffuseness parameter [*δ_γ_*] was determined using the following expression:

(1) $$\frac{\varepsilon_{\text{r,max}}}{\varepsilon_{\text{r}}}\;\;=\;\;\exp\left(\frac{{\left(T-T_{\text{m}}\right)}^{2}}{2{\delta_{\gamma }}^{2}}\right)$$

The value of *δ_γ _*was determined from a plot of ln (*ε*_r, max _*/ε_r_*) versus the (*T - T*_m_)^2 ^[16]. The values of *δ_γ _*as a function of NiO content are shown in Table 1. The parameter *δ_γ _*increased with increasing NiO content, confirming that the addition of NiO promoted the diffuse phase transition of the composites.

Huang and Tuan proposed that Ni ions could substitute the Ti ions in BaTiO_3 _lattices [17]. It has also been reported that La^3+ ^doped at the Ti site of BaTiO_3 _ceramics exhibits a change in the transition temperature as well as a pronounced diffuseness transition [18-22]. The La ions are effective in breaking the long-range order and produce Ti vacancies. This breakage of long-range ordering leads to a reduction of the ferroelectric characteristics and enhances the diffuse phase transition. In our present work, unit cell volume was calculated from XRD diffraction patterns, and the calculation result is listed in Table 1. The calculation result indicated an increase in the unit cell volume after adding NiO. This increase may be due to the Ni ions substituting the Ti ions (at the B site). Therefore, substitution of the Ni ions at the B site may result in breaking the long-range ordering, resulting in a reduction of the ferroelectric behavior with the transition becoming more diffuse [23]. Further, with increasing NiO content, the structure of the composites became more heterogeneous. This may contribute to the diffuse phase transition of the samples. From Figure 4, the increase of loss tangent with NiO content implies a higher electrical conductivity of the composites. However, the highest loss tangent in the present work was lower than 0.035, indicating that the present composites still have a potential for capacitor applications. This result also supports the reason for the presence of the lossy capacitor hysteresis behavior of the composites.

## Conclusions

In this work, the properties of BZT93/NiO composites were determined for the first time. X-ray diffraction results revealed the presence of NiO particles in the composites. The additive of NiO nanoparticles enhanced the magnetic behavior. The increase of loss tangent affected the ferroelectric hysteresis where a lossy capacitor hysteresis loop was clearly observed for the sample containing high amounts of NiO. However, the 1.0 vol.% samples showed the optimum magnetic/ferroelectric behavior. In addition, the additive also promoted the dielectric diffuse phase transition behavior while loss tangent values were still low. These characteristics of the composites may make them have potential for many electronic applications in the future.

## Competing interests

The authors declare that they have no competing interests.

## Authors' contributions

PJ carried out the experiments, analysis, and writing of the manuscript. SE, KP, and TT participated in the conception and design of the study. DPC and GR revised the manuscript for important intellectual content. All authors read and approved the final version of the manuscript.
